# Temporal Succession of Ancient Phytoplankton Community in Qinghai Lake and Implication for Paleo-environmental Change

**DOI:** 10.1038/srep19769

**Published:** 2016-01-25

**Authors:** Gaoyuan Li, Hailiang Dong, Weiguo Hou, Shang Wang, Hongchen Jiang, Jian Yang, Geng Wu

**Affiliations:** 1 State Key Laboratory of Biogeology and Environmental Geology, China University of Geosciences, Beijing 100083, China; 2 School of Earth Science and Resources, China University of Geosciences, Beijing 100083, China; 3 Department of Geology and Environmental Earth Science, Miami University, Oxford, Ohio 45056, USA; 4 State Key Laboratory of Biogeology and Environmental Microbiology, China University of Geosciences, Wuhan, 430074, China

## Abstract

Tibetan lake sediments in NW China are sensitive recorders of climate change. However, many important plankton members do not leave any microscopic features in sedimentary records. Here we used ancient DNA preserved in Qinghai Lake sediments to reconstruct the temporal succession of plankton communities in the past 18,500 years. Our results showed that seven classes and sixteen genera of phytoplankton in the lake underwent major temporal changes, in correlation with known climatic events. Trebouxiophyceae and Eustigmatophyceae were predominant during the cold periods, whereas Chlorophyceae, Phaeophyceae, Xanthophyceae, Bacillariophyceae, and Cyanophyceae were abundant during the warm periods. The inferred changes in temperature, nutrients, precipitation, and salinity, as driven by the Westerlies and summer Monsoon strength, likely contributed to these observed temporal changes. Based on these correlations, we propose the phytoplankton index as a proxy to reconstruct the stadial versus interstadial climate change history in Qinghai Lake. This taxon-specific index is free of terrestrial contamination, sensitive to short-term climatic oscillations, and continuous in recording all climatic events in the lake. The validity of this index and its applicability to other lakes is demonstrated by its good correlations with multiple climate records of Qinghai Lake and another lake on the Tibetan Plateau, Kusai Lake.

Qinghai Lake is the largest lake on the Qinghai-Tibetan Plateau in NW China, and its sediments are sensitive recorders of important paleo-climatic events^1^,^2^,^3^,^4^,^5^. Many paleo-climate proxies have revealed that the mid-latitude Westerlies and Asian summer monsoon were primary controls of late Pleistocene and Holocene temperature and hydrological changes in the lake^1^. These climatic events have likely caused changes in the general plankton ecology of Qinghai Lake and other Tibetan lakes^6^, but the temporal changes of plankton in response to climate changes are generally not well understood.

Microscopic analysis of fossil plankton preserved in lake sediments is a widely used approach in paleo-climate studies, but the majority of plankton do not have fossilizing microscopic features and are thus excluded from micro-paleontological observations. However, these non-fossilizing plankton are sensitive to environmental changes and can be useful for paleo-climate studies^7^,^8^,^9^. Fortunately, recent studies have shown that temporal changes in plankton ecology, including microscopically non-fossilizing plankton, can be reconstructed from Holocene and Pleistocene marine and lake sediments using sedimentary DNA^7^,^10^. This approach has been successfully applied to a 3100-year sedimentary record of Kusai Lake on the Tibetan Plateau^11^. Monsoon strength-driven changes in temperature, nutrient availability, and salinity were important controls for the observed temporal changes in the abundance of dominant phytoplankton.

Despite these encouraging results, it remains unclear if this ancient DNA approach can be extended into older sedimentary records on the Tibetan Plateau, such as Qinghai Lake that contains more complex plankton community than Kusai Lake^8^. Identification and quantification of individual members in a complex algal community is more difficult, and correlation of temporal variation of algal abundance with paleo-environmental change may be more challenging, because it is unclear if all algal species are responsive to such change. In addition, the climate change history in Qinghai Lake is more complex than in Kusai Lake, because the lake is located at the junction of four major climatic systems: the Westerlies, the East Asian Monsoon, the Siberian cold polar airflow, and the Indian Monsoon^1^. Multiple studies have revealed the following major paleo-climate events in Qinghai Lake: the late Pleistocene was generally cold and arid with three well-recognized cold periods corresponding to well-known climatic events Heinrich 1 (H1) or Oldest Dryas, the Older Dryas (OD), and the Younger Dryas (YD)^1^,^3^,^4^,^5^,^12^. However, some warm and moist events, e.g. the Bølling oscillation and Allerød oscillation^3^ intervened in this overall cold period. The subsequent transition to the Holocene was characterized by frequent fluctuations between warm and cold climatic phases, such as the warm Preboreal period (PP), a cooling event (CE) at 8.2 cal. kyr BP, the mid-Holocene climatic optimum (MHCO), a dry and cold period (DCP) at 4.2–2.7 cal. Kyr BP, the Medieval Warm period (MWP) at 1400 to 900 cal. yr BP, and the Little Ice Age (LIA) at 900–400 cal. yr BP^4^,^5^,^12^,^13^. During the Holocene, the 1^st^ (2000 to 1400 cal. yr BP, also called Dark Age Cold Period, DACP) and 5^th^ (7500–7600 cal. yr BP) ice rafting events were initially described from the North Atlantic region^14^, but their effects were also evident in the Qinghai Lake region^4^,^5^. The impacts of these complex climatic events on the paleoecology of Qinghai Lake have not been investigated.

In contrast to fossilized phytoplankton remains such as Bacillariophyceae (diatoms) that are widely used to quantitatively reconstruct paleo-environmental conditions^15^, insufficient attention has been given to develop new paleo-environmental proxies using non-fossilizing phytoplankton. Some organic molecule-based proxies have been developed as paleo-thermometers. For example, 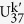 (based on alkenones produced by haptophyte algae) provides a method for reconstructing lake surface temperature^16^. However, alkenones are only produced by a few haptophytes, which are not always present in lakes, and uncertainties exist when this index is applied to lacustrine environment. Another paleo-thermometer is TEX_86_, which is based on the relative abundance of isoprenoidal glycerol dialkyl glycerol tetraethers (iGDGTs) of mesophilic marine *Thaumarchaeota*. This index was first proposed for marine system and has only recently been applied to lacustrine system but with uncertainties as well^17^. Therefore, an alternative paleoecology-based proxy is needed to reconstruct paleo-environmental conditions for lacustrine systems.

The objective of this study was to investigate the temporal succession of ancient phytoplankton community in Qinghai Lake located at the northeastern corner of the Tibetan Plateau (36°32′ - 37°15′N, 99°36′ - 100°47′E, elevation 3196 m, Fig. 1), using the sedimentary ancient DNA approach. This succession was then correlated with the paleo-environmental and paleo-climatic changes of the lake region from the late Pleistocene to the present (the past 18,500 years). These correlations can be interpreted from the inferred physiology of major phytoplankton genera and species. Based on these correlations, a potentially useful proxy, phytoplankton index, is proposed to constrain the paleo-environmental change history in Qinghai Lake. However, unlike quantitative nature of 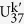 and TEX_86_, this index only provides a qualitative estimate of paleo-temperature at present (such as stadial vs. interstadial climates), largely due to lack of laboratory calibration. Finally the applicability of this index to other lakes and potential problems is discussed.

## Results and Discussion

### Chronology

The radiocarbon ages on total organic carbon of bulk sediments were converted to calendar year before 1950 (cal yr BP) using the IntCal13 calibration curve on the Calib7.0.2 program^18^ (Table 1). An exponential function was used to fit the age-depth relationship for the following three reasons: 1) this model gives a good fit with R^2^ = 0.989; 2) previous age data from Qinghai Lake also show an exponential trend (Fig. 2)^4^,^19^,^20^; 3) This model gives an age of 10,300 cal. kyr BP for the dolomite marker bed within the core, which is the same as that obtained in a previous study^4^. Our ^14^C age-depth relationship and the reservoir age of 538 years are also similar to the previously reported values of ~700 years for other cores taken from adjacent sites^3^,^4^,^19^,^20^. According to this model, the 578-cm core QH-2011 covered a time span of approximately 18,500 years with a time resolution of approximately 120 years.

### Temporal variations of paleo-limnological conditions

**Figure 1:**
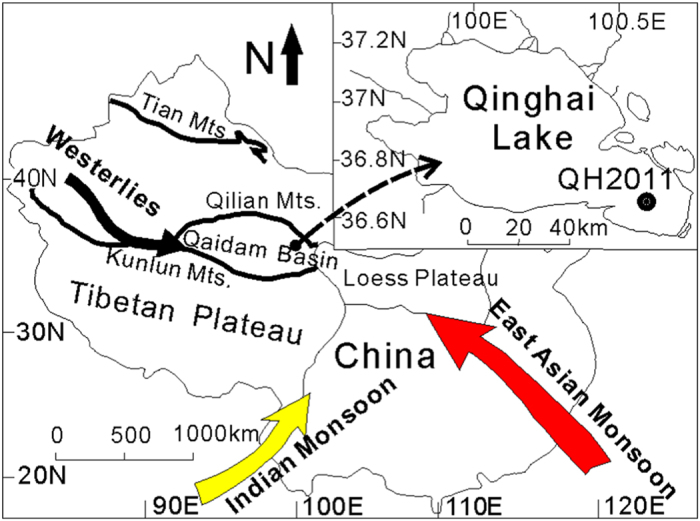
A map showing the location of Qinghai Lake on the Tibetan Plateau, the drilling site, and the climatic systems, which was modified from Dong *et al*, 2010^56^.

**Figure 2:**
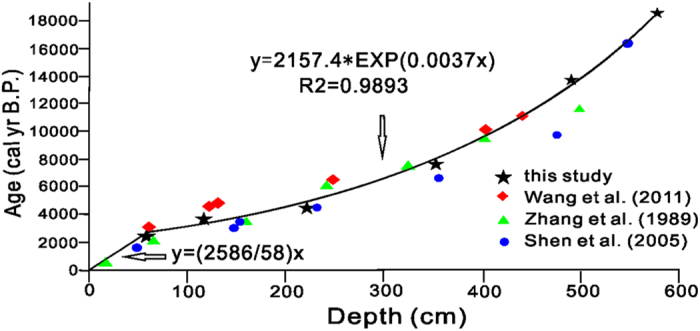
An exponential age-depth model for the Qinghai Lake sediment core (QH-2011). The ^14^C ages show a reservoir effect of about 536 years, which is in agreement with previously published reservoir effect for Qinghai Lake sediments. The overall age-depth relationship was fitted with an exponential function: y = 2157.4* e^(0.0037x)^ with an R^2^ of 0.989.

**Figure 3:**
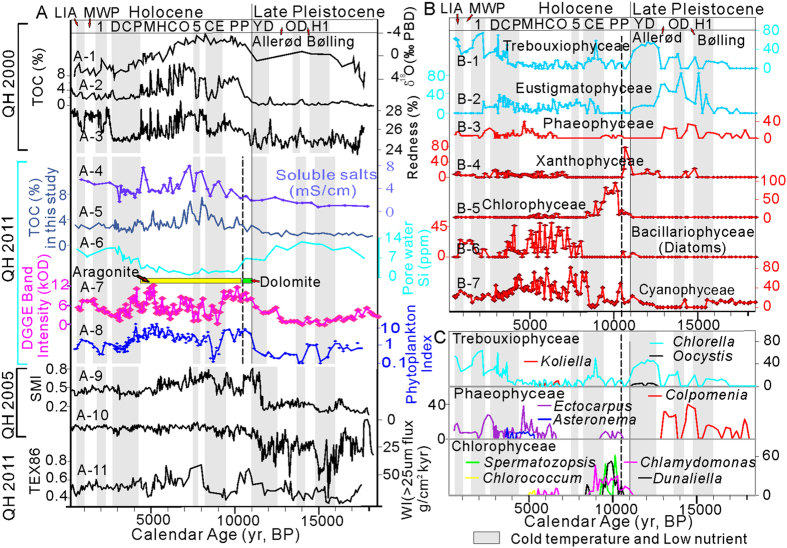
Various Qinghai Lake proxy records in the last 18,500 years. A-1 to A-3 are from the core QH-2000^4^,^5^,^12^; A4-A8 and B-1 to B-7 are from this study (QH-2011); A9-A10 are from Core QH-2005^1^; A-11 is from QH-2011^48^. A. (A-1) δ^18^O of ostracode shells of core QH-2000^12^; (A-2) Total organic carbon (TOC) of core QH-2000^4^; (A-3) Sediment redness, as measured by visible reflectance of sediment core QH-2000, is a proxy for iron oxide abundance^5^; (A-4) Concentration of soluble salts of QH-2011 sediments; (A-5) TOC of QH-2011 sediments; (A-6) Si concentration in pore water of QH-2011. The yellow and green bars between A-6 and A-7 represent aragonite and dolomite, respectively; (A-7) Total phytoplankton abundance (expressed as DGGE band intensity in unit of kilo optical density); (A-8) Phytoplankton index; (A-9 and A-10) Summer Monsoon Index (SMI) and Westerlies climate index (WI), respectively, from a previous study^1^; (A-11) TEX_86_ from a previous study^48^. The major climatic events labelled on top of this graph are: H1 - Heinrich 1 events; OD - Older Dryas event; YD - Younger Dryas event; PP - Preboreal period; CE - Cooling Event; 5 and 1–5^th^ and 1^st^ ice rafting events; MHCO – Middle Holocene climatic optimum; DCP - Dry and cold period; MWP - Medieval Warm Period; LIA – Little Ice Age. The 1st ice rafting event is also called Dark Age Cold Period (DACP). B. The relative abundances of seven major classes of the phytoplankton in Qinghai Lake sediments. C. The same plot as B but with the relative abondances of major genera shown for three classes that have multiple genera. *Fistulifera* and *Synechococcus* are the predominant genera of Bacillariophyceae and Cyanophyceae, respectively, and other genera within these two classes (Table 2) are negligible. There is only one genus within Eustigmatophyceae (*Nannochloropsis*) and Xanthophyceae (*Vaucheria*).

**Figure 4:**
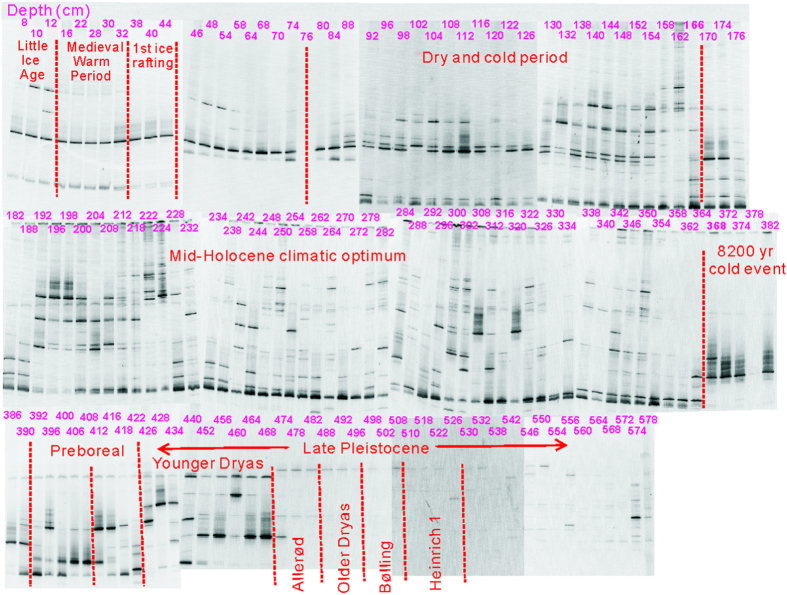
DGGE profiles of phytoplankton 23S rRNA gene fragments. The depth information (in centimeters) is given on the top of DGGE bands. The entire core (18,500 year time span) is divided into different climatic periods and they are also labeled below the depth information. Overall, there are more bands in warm periods, such as Mid-Holocene Climatic Optimum (MHCO), than in cold periods.

**Figure 5:**
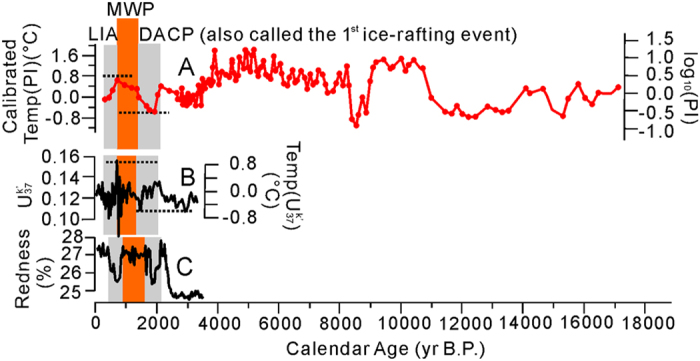
Comparison between the newly proposed phytoplankton index (**A**) and previously published 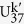
47 (B) and sediment redness^5^ (**C**) for Qinghai Lake sediments. There is only a 3500 year record available for 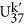 and sediment redness. Because the PI has large values, it is transformed to logarithmic function. Based on the calibration of the PI against the 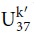 record for the first 3500 years record, the remaining temperature record (3,500 yr BP to 18.5 kyr BP) was reconstructed. The temperature was expressed as the mean annual air temperature.

**Figure 6:**
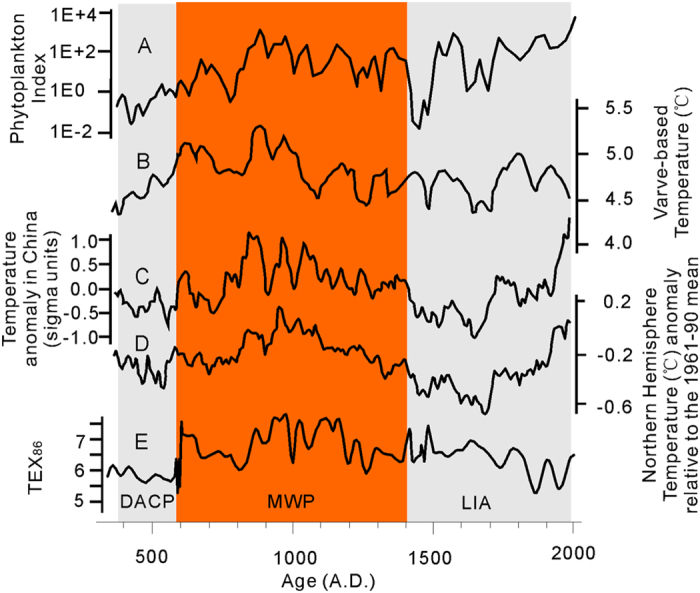
Comparison between the phytoplankton index (**A**), varve-based paleo-temperature record^49^ (**B**), temperature reconstruction of China^51^ (**C**) and the northern Hemisphere^52^ (**D**), and TEX_86_ for Kusai Lake over a 1700 year record^50^ (**E**). The varve-based paleo-temperature record is absolute temperature. The temperature reconstruction of China is anomaly relative to the average over this period. The temperature reconstruction of the North Hemisphere is anomaly relative to the average of 1961–1990. The TEX_86_ record is calculated using the composition of archaeal isoprenoidal glycerol dialkyl glycerol tetraethers (GDGTs)^50^. DACP, MWP and LIA refer to the Dark Age Cold Period, the Medieval Warm Period, and Little Ice Age, respectively^49^.

To facilitate the interpretation of temporal variations of paleo-limnological conditions in Qinghai Lake, the 18,500 year history recorded by QH2011 is divided into several periods and labeled with all known climatic events (Fig. 3). Consistent with previous studies based on the δ^18^O record of ostracode shells (Fig. 3A,A-1)^12^, total organic carbon (TOC) content (Fig. 3A,A-2)^4^, and sediment redness(a measurement of iron oxide abundance and a paleo-precipitation and paleo-temperature proxy) of a sediment core from Qinghai Lake (QH-2000, Fig. 3A,A-3)^5^, our geochemical data of core QH-2011 revealed major changes in the limonological conditions in the past 18,500 years. In particular, the concentration of soluble salts (Fig. 3A,A-4) was low in the late Pleistocene but high in the Holocene, suggesting that the Holocene is more saline and warmer than the late Pleistocene. The TOC profile from QH-2011 (Fig. 3A,A-5) was similar to the one from QH-2000^4^, with values ranging from 2% to 8%. The Si concentration in sediment pore water ranged from 0 to 14 ppm with high and low concentrations in the late Pleistocene and the Holocene, respectively (Fig. 3A,A-6). Because Si is an essential element for the growth of diatoms, the Si concentration profile should be indicative of diatom productivity. Carbonates in Qinghai Lake sediments are mainly composed of aragonite, but between 11 and 10.5 cal. kyr BP, dolomite is the dominant carbonate (yellow and green bars between A-6 and A-7 in Fig. 3A, respectively), consistent with the results of a previous study^4^. Dolomite is a common mineral in sedimentary environment, and its presence suggests a brackish and saline condition. Between the 10 and 4 cal. kyr BP, aragonite reaches the highest abundance (>40% of dry sediment weight)^4^, suggesting an overall warm period in the Holocene.

### Phytoplankton community structure in Qinghai Lake sediments

Overall there are more denaturing gradient gel electrophoresis (DGGE) bands in warm periods (such as MHCO) than in cold periods (such as late Pleistocene) (Fig. 4). Sequencing of distinct DGGE bands and phylogenetic analysis revealed that the phytoplankton community in Qinghai Lake sediments consists of at least 7 phyla, 10 classes, and 19 genera with a number of possible phytoplankton species (Table 2, Supplementary Figures S1–S5). Because the similarity values of some sequences to known culture representatives are low (85–91%), their accurate identification at genus and species level may not be possible. However, this inability should not affect our data interpretation because these genera/species only represent minor members of the phytoplankton community. Nonetheless, the growth habitat of these members can be inferred by examining the environmental conditions from which their closely related environmental sequences were retrieved (Table 2).

### Temporal succession of phytoplankton community in correlation with paleo-environmental change

DNA degradation in Qinghai Lake sediments may result in an underestimate of phytoplankton abundance, an inevitable problem in studies that use the ancient sedimentary DNA approach. However, on a relative basis, the total abundance of a phytoplankton community at a given depth can be measured with DGGE (e.g., sum of all DGGE band intensities in a given lane) and compared with those from other depths, assuming that DNA degradation does not differ between the phytoplankton members or between different sample depths. Although it has been shown that DNA preservation differs between different types of plankton (spore vs. non spore formers etc.)^21^, the broad similarity between the DGGE-derived abundance of total phytoplankton and TOC throughout this core (Compare Fig. 3A,A-4 and A-7) generally supports this assumption. Moreover, this similarity further suggests that DNA degradation did not significantly alter the major features in the depth profile of phytoplankton abundance on a relative basis, and dominant phytoplankton members are represented in DGGE bands. To further alleviate the degradation problem, in the remainder of this paper, we evaluated the relative variation of certain phytoplankton (e.g., the abundance of certain genus/species divided by total phytoplankton abundance at a given depth).

Our results show that in the past 18,500 years of the Qinghai Lake history, ancient phytoplankton communities responded to temporal variations of temperature, salinity, and nutrient level. In particular, relatively low abundance of total phytoplankton (Fig. 3A,A-7), was coincident with well-recognized cold periods including H1, OD, YD, CE, DCP, 1^st^ and 5^th^ ice-rafting events, and the LIA (Fig. 3A). Conversely, relatively high abundance of total phytoplankton coincided with warm periods including the Bølling, the Allerød, the PP, the MHCO, and the MWP. In the following sections, we divided the 18,500 years history into several periods and discussed the response patterns of 16 genera within 7 major classes (Trebouxiophyceae, Eustigmatophyceae, Phaeophyceae, Xanthophyceae, diatoms, Chlorophyceae, and Cyanophyceae) (Table 2). Although some of our following discussion is at class/genus level for the sake of clarity and conciseness, our sequence identification and inferred physiology is based on specific algal species or closely related environmental sequences.

#### Late Pleistocene (18.5– 11 cal. kyr BP)

Heinrich 1, Older Dryas, and Younger Dryas are the major climatic events during the transition from the last glacial period into the Holocene interglacial period^22^,^23^. During this period, the Qinghai Lake region was dominated by a generally Westerlies-dominated cold climate with low solar insolation and weak Asian monsoon^1^. Because Trebouxiophyceae (dominant genus *Chlorella* along with minor genera *Oocystis* and *Koliella*) and Eustigmatophyceae (genus *Nannochloropsis*) were abundant during late Pleistocene stadials (Fig. 3A–C, Table 2), they were most likely adapted to cold and oligotrophic conditions that prevailed at that time.

*Chlorella vulgaris* has been found in cold (7.6 °C) and oligotrophic South Andes lake^24^. *Chlorella* related sequences from Qinghai Lake sediments are also closely (99% similarity) related to environmental sequence (GU317418) retrieved from deep-sea (790 m, 0–4 °C) in the Pacific Ocean^25^. Likewise, psychrophilic alga *Koliella* has been found in glaciers and snow fields of Arctic, Antarctic, and alpine regions, and its nitrate reductase is highly active at 5 °C^26^. Microalgae within *Nannochloropsis* have been cultured in cold water from lakes and ponds, with a temperature of 0.6 °C^27^. *Chlorella* can survive nitrogen and phosphorus limiting conditions by enhancing lipid production^28^. Likewise, the species of *Nannochloropsis* are efficient in performing photosynthesis under long-term nitrogen stress (~30 days)^29^. These studies collectively suggest that *Chlorella*, *Koliella*, and *Nannochloropsis* related algal species can tolerate and adapt to both cold and oligotrophic conditions.

During short-term warm intervals (Bølling and Allerød oscillations) of this overall cold period, the relative abundances of presumably cold-adapted Eustigmatophyceae and Trebouxiophyceae decreased, but those of warm-adapted Phaeophyceae (i.e., *Colpomenia*), Xanthophyceae, and Cyanophyceae increased (Fig. 3B,C). Under laboratory culture condition, the growth rate of *Colpomenia* was low at low temperature (5 °C), but significantly higher at 13 or 20 °C^30^.

#### The Preboreal period (11–9.3 cal. kyr BP)

This period is the first stage of the Holocene epoch when the temperature and precipitation notably increased worldwide, primarily because of increased Asian summer Monsoon^4^,^31^. In the Qinghai Lake region, this period was characterized by a warmer climate with higher precipitation than the late Pleistocene, as evidenced by higher δ^18^O values of ostracode shells^12^, higher TOC^4^, higher sediment redness^5^, and higher carbonate content in Qinghai lake sediments (Fig. 3A). During this intensified Asian summer monsoon period, a high amount of precipitation may have carried terrestrial nutrients into the lake. However, warm temperature also would result in high evaporation, and therefore the salinity of lake may have increased^32^. According to the temperature and salinity temporal variation patterns, this period can be divided into two sub-periods (Preboreal I & Preboreal II). The Preboreal period I (11,000 to 10,500 cal. yrs BP) is characterized by a warm and brackish environment as indicated by the occurrence of a large amount of dolomite (40%, Fig. 3A). The Preboreal period II (10,500 to 9,300 cal. yrs BP) is characterized by a warm climate as indicated by previously reported aragonite^3^,^4^.

Corresponding to these paleo-limnological changes, the phytoplankton community composition shifted from the Pleistocene to the Preboreal period: presumably cold-adapted genera of classes Trebouxiophyceae and Eustigmatophyceae dramatically decreased in their abundance and even disappeared. Instead, Xanthophyceae (*Vaucheria litorea*) dominated the Preboreal period I, and Chlorophyceae (*Chlamydomonas*, *Dunaliella parva*, and *Spermatozopsis*) dominated the Preboreal period II with some occurrences of diatoms and Cyanophyceae (Fig.3B,C and Table 2).

*Vaucheria litorea* is frequently encountered in salt-marshes and salt flats^33^, grows well in up to 50% salinity, and is believed to be resistant to desiccation^34^. Laboratory experiment has shown that the growth and reproduction of several species of *Vaucheria* is favored under conditions of moderate temperatures (15°–20 °C)^35^. These physiological characteristics probably account for its dominance in a warm and brackish environment from 11000 to 10500 cal. yrs BP (i.e., the Preboreal period I) when dolomite precipitated (Fig. 3A).

Parallel to the disappearance of dolomite and emergence of aragonite in the Preboreal period II, presumably halophilic and warm *Vaucheria litorea* is replaced by *Chlamydomonas*, *Dunaliella parva*, and *Spermatozopsis*. *Chlamydomonas* is a genus of green algae and is generally found in warm (optimal temperature 23 ^o^C) and nutrient-rich habitats^36^. *Dunaliella parva* is a putatively thermo-resistant species (optimal growth temperature 31 °C)^37^ and can possibly tolerate a high salinity (31–350%) by accumulating large amounts of intracellular glycerol^38^. UV-sorbing pigment such as scytonemin allows *Dunaliella* sp. to survive under strong radiation^38^ and possibly in high elevation such as Qinghai Lake. Likewise, *Spermatozopsis* is putatively a warm algal genus with a growth temperature range of 10–15 ^o^C^39^. All these genera show a high degree of variability in their abundance during the Preboreal period II, which appears to be related to climatic oscillations.

These results imply that the warm climate, increased salinity, and high nutrient level during the Preboreal period were favorable for the growth of presumably warm and saline Xanthophyceae and Chlorophyceae, but unfavorable for the growth of putatively cold-adapted Eustigmatophyceae and Trebouxiophyceae.

#### A cooling event (9.2–8.2 cal. kyr BP)

An abrupt cooling event occurred around 8,200 years ago in both North Atlantic and Asia, and lasted for different times in different places^4^,^40^. In the Qinghai Lake region, this event lasted from ~9200 to 8200 yrs BP^4^. As a response, the phytoplankton community in Qinghai Lake shifted from a presumably warm temperature and high salinity adapted community (e.g., Xanthophyceae and Chlorophyceae) back to a putatively cold-adapted and oligotrophic Eustigmatophyceae and Trebouxiophyceae community (Fig. 3B,C).

#### Middle Holocene Climate Optimum (8.2–4.2 cal. kyr BP)

The middle Holocene is a warm, high evaporation and saline period, and is often called the Middle Holocene Climate Optimum^4^,^5^,^7^. During this period, diatoms and Cyanophyceae (mainly *Synechococcus*) emerged as dominant members of the phytoplankton community in Qinghai Lake, whereas the presumably cold-adapted Eustigmatophyceae and Trebouxiophyceae decreased in their abundances (Fig. 3B). Diatoms and Cyanophyceae have requirements for warm temperature, high nutrient levels, and a certain level of salinity^41^,^42^,^43^. Indeed, a recent study showed that the contribution of diatoms to water column productivity in oceans is positively correlated with sea surface temperature^42^. *Fistulifera* sp. JPCC DA0580, the dominant species of diatoms in Qinghai Lake, grows well from 20 to 35 ^o^C^44^ in nutrient-rich rivers and coastal waters with a high salinity (optimum growth of 16–23% salinity)^45^. The *Fistulifera* related sequences in Qinghai Lake are closely (99% similarity) related to an environmental sequence (EU342146) retrieved from a tropical stream periphyton community. Likewise, the contribution of Cyanobacteria to water column primary productivity has been shown to be positively correlated with sea surface temperature (optimal growth temperature 27 ^o^C)^42^,^46^. *Synechococcus*, the dominant cyanobacterial species in Qinghai Lake sediments, is typically marine and its abundance has been shown to positively correlate with salinity^43^.

#### Dry and cold period (4.2–2.7 cal. kyr BP)

Multiple proxies from Qinghai Lake sediments have documented this dry and cold period (Fig. 3A)^5^. In this period, the abundances of presumably cold-adapted Eustigmatophyceae and Trebouxiophyceae increased again, but the abundances of the other five putatively warm phytoplankton classes all decreased. The weakening of the Asian summer monsoon strength may have resulted in a decrease of lake temperature and a decline of terrestrial nutrient inputs during this period. These conditions were favorable for the growth of Eustigmatophyceae and Trebouxiophyceae, but unfavorable for the growth of warm and saline algae.

#### The last 2.7 kyr of Qinghai Lake history

This period is characterized by a rapid rise of temperature and precipitation, and increase of nutrients in Qinghai Lake but interrupted by some short-lived cold events such as the 1^st^ ice rafting event and the LIA (Fig. 3A)^5^. As a response to this overall warming trend, the abundances of presumably cold and/or oligotrophic genera of Eustigmatophyceae and Trebouxiophyceae decreased, but those of putatively warm and eutrophic Phaeophyceae and diatoms increased (Fig. 3B,C). The Trebouxiophyceae thrived during the 1^st^ ice rafting period. During the MWP, the abundance of Trebouxiophyceae decreased dramatically, but the abundances of presumably warm diatoms and Cyanophyceae increased (Fig. 3B,C). During the LIA, the Trebouxiophyceae once again replaced the diatoms and Cyanophyceae.

In summary, climate-driven limnological changes in temperature, precipitation, nutrient level, and salinity all contributed to the observed temporal changes in the paleo-phytoplankton community structure and abundance in Qinghai Lake in the last 18,500 years. The dominant algae in Qinghai lake sediments can be broadly classified into two categories: presumably cold and warm adapted genera/species. Because these two types of algae exhibited opposite patterns to the same paleo-limnological changes, we argue that it is necessary to examine their individual response patterns for ecology-based paleo-climate reconstruction.

### Phytoplankton index as a potential new proxy for paleo-temperature

Based on our observations that the relative abundance of presumably cold- and warm-adapted phytoplankton is correlated with stadial and inter-stadial period, respectively, we propose the phytoplankton index (PI) (Fig. 3A,A-8) as a potential paleoecology proxy for paleo-climatic change. We define the PI as follows:


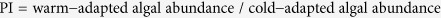


The temporal variation of the PI can be correlated with all climatic events (e.g., H1, OD, YD, PP, CE, MHCO, and DCP). In comparison with other paleo-climate proxies, this index displays certain advantages. For example, the summer monsoon index (Fig. 3A,A-9) does not display much climate variability in the late Pleistocene, whereas the Westerlies index (Fig. 3A,A-10) does not reveal important climatic events in the Holocene. However, the PI reveals all climatic events in both periods. Furthermore, the PI distinctly reveals the two short warm periods in the overall cold late Pleistocene period: the Allerød and Bølling oscillations^3^. Remarkably, the PI also reveals climatic oscillations during the pre-boreal period (Fig. 3A,A-8), which implies that the climatic transition from the cold Pleistocene to the warm Holocene was not unidirectional. These climatic oscillations were observed previously^32^ and revealed by our geochemical data, e.g., the occurrence of dolomite in the Preboreal period I and its disappearance in the Preboreal period II (Fig. 3A). In addition, the PI is also sensitive to other short-lived climatic events such as the LIA, and 1^st^ and 5^th^ ice rafting events. The high sensitivity of the PI to these short-lived events suggests that the phytoplankton community in Qinghai Lake responded quickly to paleo-environmental changes, likely due to their surface water growth habitat^16^ and short generation time.

Previous studies have used and 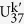
^47^ and TEX_86_^48^ to quantitatively reconstruct Qinghai Lake paleo-temperature records. Paleo-temperature record for Qinghai Lake is also available indirectly through sediment redness^5^. Therefore, the newly proposed PI can be compared with these previously tested records. In general, the PI (Fig. 5A) is in good agreement with records of 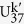 (Fig. 5B) and sediment redness (Fig. 5C) over the last 3500 years. Specifically, the PI curve shows a rapid cooling at the onset of the 1^st^ ice rafting event, similar to the sediment redness curve, but a gradual warming at the onset of the MWP, similar to the 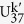 record. Based on the calibration of our PI record against 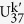 and sediment redness records for the first 3,500 years (e.g., the highest temperature of 0.8 °C at the MWP and lowest temperature of 0.6 °C at the 1^st^ ice rafting event), the entire 18,500 year temperature record of Qinghai Lake could be reconstructed (Fig. 5A). Similar to the 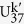 record, this reconstructed record represents mean annual air temperature of Qinghai Lake water.

The PI is also in agreement with the TEX_86_ index (Fig. 3A,A-11), but with a higher sensitivity. For example, from 10000 to 8000 yr B.P., the TEX_86_ index shows a long and stable cold period, but the PI shows some oscillations, suggesting that the phytoplankton may be more sensitive to temperature than pelagic archaea, which is the basis for TEX_86_. Alternatively, plankton communities may have changed as a result of environmental changes other than temperature, because there were no obvious changes in temperature recorded from TEX_86_ in that time frame.

### Validation of phytoplankton index in Kusai Lake

To further validate the PI, we compiled several paleo-temperature proxies for Kusai Lake, another saline lake on the northern Tibetan Plateau (Fig. 6). In this lake, the phytoplankton community is simpler than in Qinghai Lake and is only dominated by warm adapted *Synechococcus* and cold adapted *Isochrysis*^11^. So the PI is calculated by the abundance ratio of these algal species (Fig. 6A). Independent temperature proxies are available for the past 1700 years based on annual varve thickness^49^ and TEX_86_^50^. All these three datasets were obtained from the same sediment core (5 m long), where no evidence of biodegradation was found over this short time interval^50^. Liu *et al.* (2014) demonstrated that the thickness of light-colored varve layer is positively correlated with temperature, because warm summers can often result in high runoff and sediment flux to the lake, forming thick and light sediment layers with clay minerals and carbonates as dominant minerals^49^. The varve-based paleo-temperature record in Kusai Lake (Fig. 6B) was validated by comparisons with the annual summer temperature of the 1959–2007 A.D. recorded by the nearby Wudaoliang station and with the tree-ring records from Karakorum of Pakistan (600 to 2000 A.D.). This varve-based temperature record also compares favorably with temperature construction of China^51^ (Fig. 6C) and the Northern Hemisphere (Fig. 6D)^52^. The TEX_86_ record was calculated based on the relative abundance of Thaumarchaeota-biosynthesized iGDGTs in the same sediment core (Fig. 6E), but its temporal resolution (~20 years) is much lower than the annual temperature record at the nearby Wudaoliang station. The TEX_86_ is thus not calibrated against temperature.

The PI record shows a similar trend of temperature variation to all three well-established temperature records, including varve-based temperature record for Kusai Lake (r = 0.185, p = 0.037, n = 127), temperature reconstructions of China (r = 0.328, p = 0.001, n = 127), and the northern Hemisphere (r = 0.201, p = 0.023, n = 127), demonstrating the reliability of this newly proposed index in paleo-temperature reconstruction (Fig. 6A–D). The PI record also shows a similar structure to the TEX_86_ record for Kusai Lake (Figs. 6A,E). All three temperature proxies, including the PI, varve-based temperature record, and the TEX_86_, show a consistency in the DACP and the MWP. However, some differences were observed in the LIA period. Whereas the PI record shows an abrupt drop in temperature at the end of the MWP and the beginning of the LIA (Fig. 6A), and the temperature reconstructions of China (Fig. C) and the Northern Hemisphere (Fig. 6D) show a more gradual cooling trend^52^. The varve-based temperature does not show a decrease in temperature except for a few short cooling intervals (Fig. 6B). In addition, unlike the rapid response of the algae-based PI to the LIA at ~1400 A.D., the archaea-based TEX_86_ record does not show this response until ~1800 A.D. This lag time of 400 years suggests that archaea is slower than algae in responding to the same climatic event. A similar lag in the response time of archaea (e.g., 400 years) has been reported for Kusai Lake, based on measured changes of iGDGT composition^50^. Collectively, these comparisons suggest that past phytoplankton communities and therefore the PI may be more sensitive to environmental change than varve thickness or archaeal lipid based paleo proxies.

For a wide application of the PI to other lakes, it is necessary to quantify possible algal genera/species and to classify them into warm/cold categories. The cyanobacteria and eukaryotic algae detected in Qinghai Lake sediments are common genera/species that should be widely present in many lakes. Many of these algal species have specific lipid biomarkers (Castaneda and Schouten 2011) that can be used to rapidly identify and quantify their relative abundance. In case no genera/species specific lipid biomarkers are available, our sedimentary DNA-based approach is necessary to be able to calculate the PI. Our DGGE and sequencing based approach provides a quick and fairly inexpensive method for the calculation. However, in case of a complex algal community, quantitation of individual members in the phytoplankton community and classification of their growth habitats require efforts. Fortunately, rich algal databases are available to accomplish these tasks. Future studies can be performed to quantitatively calibrate the abundance of the observed algae to environmental conditions using surface sediments or time series across multiple lakes. We recognize that paleo-limnological conditions are complex, and multiple environmental factors may simultaneously influence a phytoplankton community. In this case, this index may reflect the broad importance of stadial vs. interstadial climates, rather than specific environmental conditions such as temperature.

## Materials and Methods

Qinghai Lake (36°32′ to 37°15′N, 99°36′ to 100°47′E) is a perennial lake located at the northeastern corner of the Tibetan Plateau with an elevation of 3196 m above sea level (Fig. 1). The lake is located in a structural intermontane depression at the northeastern corner of the Tibetan Plateau (Fig. 1). The lake has a surface area of 4300 km^2^ and lies within a catchment of limestone, sandstone, and shale. The average water depth is 19.2 m with the maximum of 28.7 m. The amount of evaporation of the lake (~1400 mm/year) is in excess of the mean annual precipitation (~400 mm/year), resulting in a mesohaline lake. Qinghai Lake is separated into two sub-basins by a normal faulting horst in the middle of the lake. The northern sub-basin is more dynamic than the southern sub-basin because of riverine input in the north.

In August 2011, a 578-cm long sediment core (QH2011) was retrieved from the southeastern corner of the southern sub-basin (Fig. 1) at a water depth of 24 m using a coring platform. After retrieval, the core was cut into 30–40 cm segments and their ends were sealed with sterilized plastic lids. The core segments were kept in dry ice during transportation and stored at −80 °C upon arrival in the laboratory until analysis. The frozen core segments were dissected into 2-cm slices with a total of 289 subsamples. Approximately every other sub-sample (154 out of 289 slices) was used for subsequent geochemical and molecular analyses.

Because Qinghai Lake sediments do not contain any terrestrial plant macrofossils, bulk organic carbon was used for accelerator mass spectrometry (AMS) ^14^C dating. AMS ^14^C ages from six depths of core QH2011 were determined at Beta Analytic Inc. (Miami, Florida, USA). The samples were pre-treated with 1 N HCl to remove any inorganic carbon (carbonates). The treated samples were first measured for TOC and total nitrogen to ensure that an adequate amount of sample was used for ^14^C dating.

Sixty-six sediment sub-samples were used for sediment soluble salt content and pore water chemistry following separation of pore water from sediments by centrifugation. Ten grams of air-dried sediment subsamples were analyzed for concentrations of soluble salts at the Service Testing and Research (STAR) lab of the Ohio State University, USA, according to previously published methods^53^.

Anion concentrations of pore water samples were determined using high-performance liquid chromatography (HPLC, Dionex DX-500 chromatography, Dionex Co.), and cation concentrations were determined using direct current plasma optical emission spectrometry (DCP-OES, Beckman).

One hundred and one sediment subsamples were analyzed for TOC after removal of inorganic carbon with 1N hydrochloric acid and rinse with deionized water (4 times). After drying, TOC content of the subsamples was measured using a multi N/C 2000 analyzer (multi N/C® 2000, Germany) with furnace temperature of 1000 °C.

According to a previous study, the predominant carbonate minerals in Qinghai Lake sediments are aragonite and dolomite^4^. Here we investigated the abundances of these carbonate minerals in 22 subsamples with quantitative X-ray diffraction (qXRD). Corundum was used as an internal standard. In order to avoid any orientation effect of clay particles, powdered sediment subsample was side-packed into a quartz sample holder. Randomly oriented sample was X-ray scanned from 3 to 70 degree two theta with Cu K-alpha radiation (40 kV, 35 mA), a 0.02 degrees step size, and a count time of 1 s per step. XRD patterns were analyzed quantitatively and the weight percent of each mineral was obtained using the Rock-Jock computer program^54^.

Genomic DNA was extracted from 154 sediment sub-samples (~0.5 g per sample) with FastDNA SPIN Kit for Soil (MP Biomedicals, USA) in a laminar flow hood that was thoroughly sterilized with ultraviolet radiation for 30 min and 6% sodium hypochlorite according to a previously published protocol^11^. The hood was placed in a dedicated room designed for ancient DNA isolation. A blank control was included during DNA extraction and sequencing. The extraction quality was ensured according to a previously published protocol^11^. DNA concentration was quantified by measuring optical absorbance at 260 nm using a NanoDrop ND-1000 spectrophotometer (Thermo Scientific, Wilmington, Delaware, USA) and an average from triplicate measurements was reported.

To determine the abundance and diversity of ancient phytoplanktonic communities preserved in Qinghai Lake sediments, the homologous 23S rDNA fragments of both cyanobacteria and chloroplast of eukaryotic algae were amplified for 154 sediment subsamples with polymerase chain reaction (PCR) using the GC-clamped specific primers p23SrV_f1-GGACAGAAAGACCCTATGAA/p23SrV_r1-TCAGCCTGTTATCCCTAGAG^55^. The PCR product was then run with DGGE^11^. DGGE is a form of electrophoresis which uses a chemical gradient to denature a DNA sample as it moves across an acrylamide gel. DGGE banding patterns can be used to visualize variations in microbial genetic diversity and band intensity provides a rough estimate of abundance of predominant microbial community members. The genetic marker of 23S rDNA (410 base pairs) was used in this study because it is present in both prokaryotic cyanobacteria and eukaryotic algae, and can be used to compare their relative abundances. The PCR products were separated by DGGE, and distinct bands were excised, re-amplified with the same primer set but without the GC clamp, and sequenced^11^. The 23S rDNA sequences were taxonomically assigned to specific genera and species using the Basic Local Alignment Search Tool (BLAST) in the NCBI database ( http://www.ncbi.nlm.nih.gov). Neighbor-joining trees were constructed to show the phylogenetic relationships between the planktonic 23S rDNA sequences obtained in this study to their closely related relatives from the GenBank database. The relative abundance of individual phytoplankton classes/genera/species was quantified by dividing the total intensity of all bands that belong to that class/genus/species by the total intensity of all classes/genera/species and expressed as relative percentages. This relative abundance approach makes it possible to compare algal abundance across different taxa, assuming that DNA degradation occurred uniformly to all algal genera/species. DGGE band intensity was quantified using the Quantity One® software. The sequences obtained in this study were deposited in the NCBI database under accession numbers KF803784–KF803988.

## Additional Information

**How to cite this article**: Li, G. *et al.* Temporal Succession of Ancient Phytoplankton Community in Qinghai Lake and Implication for Paleo-environmental Change. *Sci. Rep.* **6**, 19769; doi: 10.1038/srep19769 (2016).

## Supplementary Material

Supplementary Material

## Figures and Tables

**Table 1:** ^14^C AMS ages analyzed on total organic carbon (TOC) and calibrated ages for Qinghai Lake.

Depth(cm)	Conventional ^14^C age ± σ/yr, BP	Reservoir-corrected 14C age by 538 yr/yr, BP	Calendar age based on 2σ/cal yr BP		Median of two calendar ages/cal yr BP
58	3020 ± 30	2482	2453	2719	2586
116	4050 ± 30	3512	3699	3866	3783
222	4540 ± 30	4002	4418	4526	4472
352	7300 ± 40	6762	7571	7674	7623
488	12530 ± 50	11992	13726	13995	13861
576	15770 ± 80	15232	18454	18685	18570

**Table 2:**
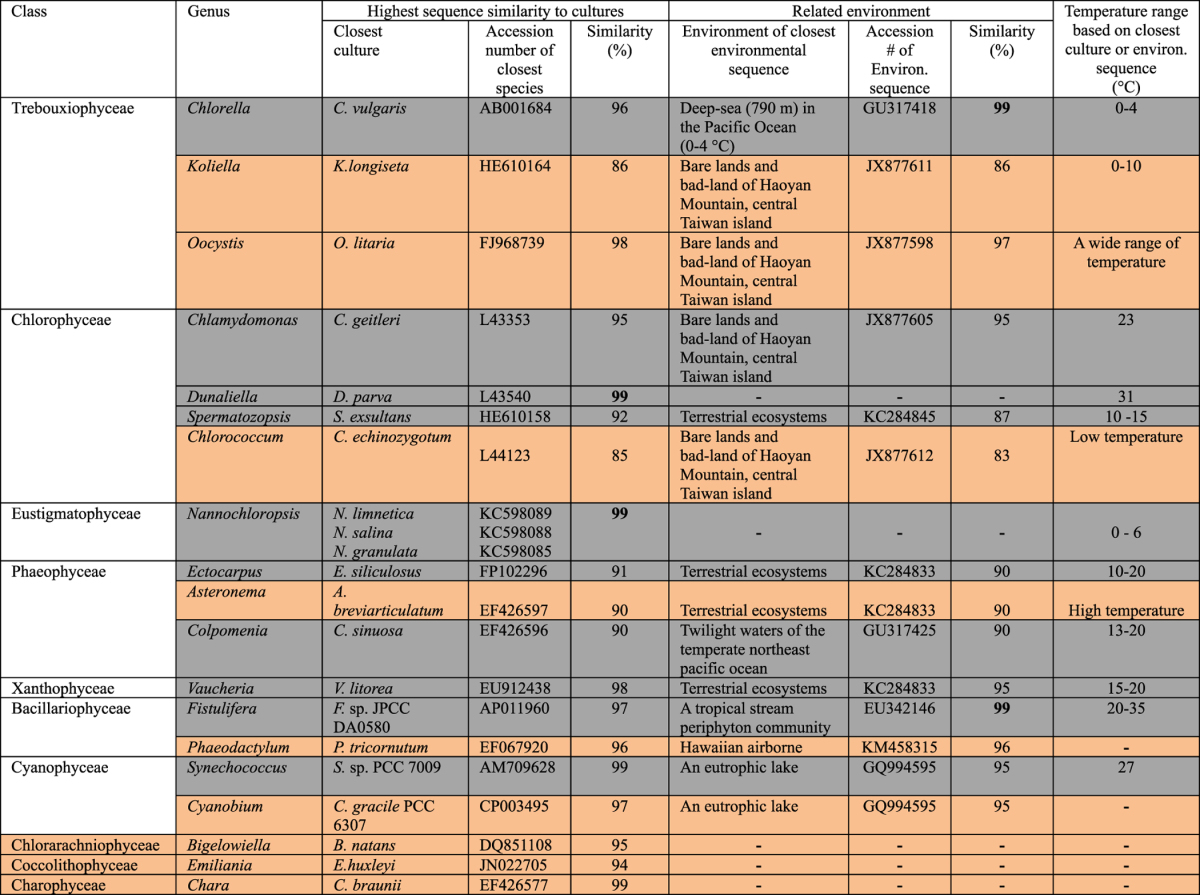
Total phytoplanktonic community in Qinghai Lake sediments.

Orange and grey color denotes minor and major phytoplanktonic members respectively.
