# Supplementary material for: Temporal Succession of Ancient Phytoplankton Community in Qinghai Lake and Implication for Paleo-environmental Change
Source: Sci Rep. 2016 Jan 25;6:19769. doi: 10.1038/srep19769 (PMC4726407; doi:10.1038/srep19769)
Supplement: Supplementary Material [file srep19769-s1.doc]

**Temporal Succession of Ancient Phytoplankton Community in Qinghai Lake and**

**Implication for Paleo-environmental Change**

Gaoyuan Li^1,2^, Hailiang Dong^1,2,3*^, Weiguo Hou^1*^, Shang Wang^1,2^, Hongchen Jiang^4^, JianYang^4^, and Geng Wu^4^

*^1^State Key Laboratory of Biogeology and Environmental Geology, China University of Geosciences, Beijing 100083, China;*

*^2^School of Earth Science and Resources, China University of Geosciences, Beijing 100083, China;*

*^3^Department of Geology and Environmental Earth Science, Miami University, Oxford, Ohio 45056, USA;*

*^4^State Key Laboratory of Biogeology and Environmental Microbiology, China University of Geosciences, Wuhan, 430074, China*

* Corresponding authors: Hailiang Dong and Weiguo Hou

*State Key Laboratory of Biogeology and Environmental Geology*

*China University of Geosciences, Beijing 100083, China*

Email: [dongh@cugb.edu.cn](mailto:dongh@cugb.edu.cn), [dongh@miamioh.edu](mailto:dongh@miamioh.edu), or weiguohou@cugb.edu.cn

Revised for Scientific Reports

September 9, 2015

**Supplementary Figures**

Figure S1. Neighbor-joining tree showing the phylogenetic relationship among all phytoplankton 23S rRNA gene fragments retrieved from DGGE bands and their closely related sequences from GenBank. The phylogenetic tree was constructed with the neighbor-joining method. The scale bar indicates the Jukes-Cantor distance. Bootstrap values (1000 replicates) of > 50% are shown.

Figure S2. The unfolded neighbor-joining tree of Group a from Fig. S1 (*Eustigmatophyceae*).

Figure S3. The unfolded neighbor-joining tree of Group b from Fig. S1 (*Xanthophyceae*).

Figure S4. The unfolded neighbor-joining tree of Group c from Fig. S1 (*Bacillariophyceae*)

Figure S5. The unfolded neighbor-joining tree of Group d from Fig. S1 (*Cyanophyceae*)
